# Novel Proton Conducting Solid Bio-polymer Electrolytes Based on Carboxymethyl Cellulose Doped with Oleic Acid and Plasticized with Glycerol

**DOI:** 10.1038/srep27328

**Published:** 2016-06-06

**Authors:** M. N. Chai, M. I. N. Isa

**Affiliations:** 1 Advanced Materials Team, Ionic State Analysis (ISA) Laboratory, School of Fundamental Science, Universiti Malaysia Terengganu, Kuala Terengganu 21030, Terengganu, Malaysia

## Abstract

The plasticized solid bio-polymer electrolytes (SBEs) system has been formed by introducing glycerol (Gly) as the plasticizer into the carboxymethyl cellulose (CMC) doped with oleic acid (OA) via solution casting techniques. The ionic conductivity of the plasticized SBEs has been studied using Electrical Impedance Spectroscopy. The highest conductivity achieved is 1.64 × 10^−4^ S cm^−1^ for system containing 40 wt. % of glycerol. FTIR deconvolution technique had shown that the conductivity of CMC-OA-Gly SBEs is primarily influenced by the number density of mobile ions. Transference number measurement has shown that the cation diffusion coefficient and ionic mobility is higher than anion which proved the plasticized polymer system is a proton conductor.

Recently, biodegradable materials attract enormous attention worldwide as a result of white pollution, one of the environmental crises. Several renewable resource-based biopolymers are suitable to be used as host polymer in the polymer electrolytes^1^,^2^,^3^, such as starch^4^, cellulose^5^,^6^,^7^,^8^, chitosan^9^,^10^,^11^,^12^, carrageenan^13^,^14^ and agarose^15^,^16^. The polymers can solvate the dopant if there is direct interaction between the lone pair electron of the heteroatom such as oxygen or nitrogen in the polymer and cation of the ionic dopant^2^,^17^. Therefore, it is a significant develop solid biopolymer electrolytes (SBE) by using natural polymer.

Cellulose-based solid polymer electrolyte have received much attention over the past few years for many applications such as batteries, fuel cells, super capacitors, display devices, sensors, etc.^3^,^17^,^18^. Due to its high degree of crystalline, cellulose-based polymer electrolyte faced an inherent problem of low ionic conductivity that limits the application of this type of polymer electrolyte^19^,^20^,^21^. We have reported the effect of ionic dopant i.e. oleic acid (OA) on carboxymethyl cellulose (CMC)^22^. In order to enhance the ionic conductivity, the addition of plasticiser was studied in this work. According to previous researches^23^,^24^,^25^, plasticizers would turn the texture of polymer to become softer and more flexible, and enhance the chemical and mechanical stability of membranes since they could penetrate and increase the distance of molecules and decrease the polar groups of polymer.

In this present work, glycerol (Gly) was chosen as the plasticizer in order to increase the ionic mobility of the materials and hence elevate the conductivity of the CMC- 20 wt. % OA system^22^. The findings in this research offer a new possibility and provide educators and researchers on the significant effects of diverse concentrations of Gly on CMC-OA SBE’s conductivity. Furthermore, details analysis on the ionic transport properties via FTIR-deconvolution technique open-up to new insights on the conduction behaviour of the plasticized bio-based materials.

## Methods

### Development of CMC based solid bio-polymer electrolytes

The development of CMC-OA SBEs is following the previous work done reported in ref. 22. 1 g of CMC was dissolved in distilled water while 0.25 g of oleic acid was dissolved in ethanol (solvent) in separate beaker before combining both solutions. Then different weight percentage (wt. %) of Gly was incorporate into the mixed solution and stirred until it dissolved completely with no phase seperation. The final clear solution was then cast into separate Petri dishes and dried in the oven at 60 °C. SBE films were transferred to desiccators for further drying prior to characterization. The composition of the samples and their designation are tabulated in Table 1. A control sample of CMC with 20 wt. % OA–0 wt. % Gly was also prepared for comparison.

### Characterization of CMC based Solid bio-polymer electrolytes

#### Conductivity Study

The SBE films was analyse via Electrical Impedance Spectroscopy model HIOKI 3532-50 LCR Hi-Tester at varies frequency of 50 Hz to 1 MHz. The films was cut into a fitting size of circle with diameter 2 cm and placed between two stainless-steel blocking electrodes of the sample holder which connected to the LCR tester. The software controlling the measurement recorded the real and imaginary impedance at various frequencies. The bulk impedance (*R*_*b*_) value was obtained from the plot of negative imaginary impedance (−*Z*_*i*_) versus real part (*Z*_*r*_) of impedance and the conductivity of the samples were calculated as follow^9^,^11^:


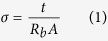


where *A* = area of SBE–electrode contact and *t* = thickness of the SBE films.

#### Fourier Transform Infrared (FTIR) spectroscopy

A Thermo Nicolet Avatar 380 FTIR spectrometer was used to analyse the SBE films. The spectrometer was equipped with an attenuated total reflection (ATR) accessory with a germanium crystal. The sample was put on a germanium crystal and infrared light was passed through the sample with a frequency ranging from 4000 to 675 cm^−1^ with spectra resolution of 4 cm^−1^.

### FTIR Deconvolution Study

The FTIR deconvolution technique was based on the work done by^26^ where the Gaussian–Lorentz function is adapted to the Origin Lab software. In the deconvolution technique, the FTIR peaks due to the dominant ionic movement were selected and the sum of the intensity of all the deconvoluted peaks was ensured to fit the original spectrum^26^.

The area under the peaks was determined and the percentage of free ions was calculated using the equation below^26^:


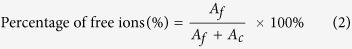


Here, *A*_*f*_ is the area under the peak representing the free ions region and *A*_*c*_ is the total area under the peak representing the contact ions.

The number density (*n*), mobility (*μ*) and diffusion coefficient (*D*) of the mobile ions were calculated following equation:


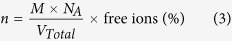



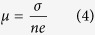



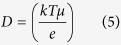


where *M* is the number of moles of dopant used in each electrolyte, *N*_*A*_ is Avogadro’s number (6.02 × 10^23^ mol^−1^), *V*_*Total*_ is the total volume of the SBEs, *σ* is dc conductivity, *e* is the electric charge (1.602 × 10^−19^ C), *k* is the Boltzmann constant (1.38 × 10^−23^ J K^−1^) and *T* is the absolute temperature.

#### Ionic Transport Study

Transference number measurements (TNM) were performed to show the relationship between the diffusion of ion to the conductivity behaviour of CMC-OA-Gly SBEs. The technique used is dc polarization^27^. The transference number (*t*_*ion*_) was determined by monitoring the current as a function of time on application of a fixed dc voltage (1.5 V) across the sample sandwiched between two stainless steel electrodes. The diffusion coefficients of cations and anions in each of the CMC-OA-Gly SBEs were calculated from the measured values of conductivity and cation transference number (*t*_+_) according to the following equations^27^,^28^:


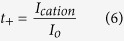



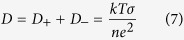



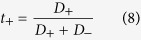


Besides, the ionic mobility can be defined according to the following equation^27^,^28^:


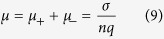



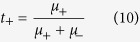


where, *μ*_+_ and *μ*_*−*_ is the ionic mobility of cation and anion.

## Result and Discussion

### Conductivity Analysis

**Figure 1:**
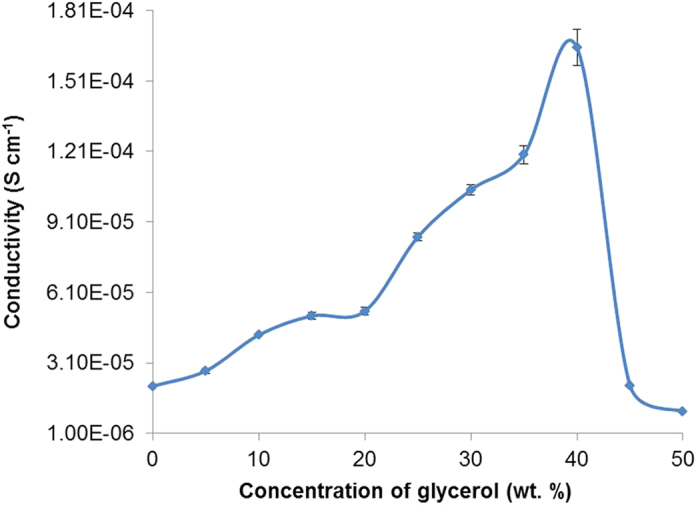
The conductivity of CMC-OA-Gly SBEs at room temperature.

The conductivity graph of CMC-OA-Gly SBEs (Fig. 1) showed that the conductivity of the CMC-OA-Gly SBEs has increases upon the addition of the Gly plasticizer. It increases from 2.11 × 10^−5^ S cm^−1^ (OA-20) to 1.64 × 10^−4^ S cm^−1^ (Gly-40). The increase in conductivity of the CMC-OA-Gly SBEs is due to the decrease of bulk resistance in the system similarly found by other workers^29^,^30^,^31^.

By postulating the existence of separate ionic pathways for the migration of free ions through the plasticizer, it is possible to explain the improvement of the conductivity by the addition of Gly and the dependence of the conductivity on the plasticizer concentrations^20^,^21^. When the amount of Gly is increased, the ions would transport mainly in the plasticizer-rich phase^23^. Gly increases the dissociation of ionic dopant and thereby produces free ions which further proven in FTIR deconvolution analysis. The effect of the Gly on the SBEs mobility and conductivity depends on the specific nature of the plasticizer including viscosity, dielectric constant, polymer plasticizer interaction, and ion plasticizer coordination^29^,^30^.

As we have reported previously^25^,^32^, the relationship between conductivity and temperature of the SBEs are naturally Arrhenius behaviour. The thermal properties results of CMC-OA-Gly SBEs shows a good fit of *R*^*2*^ ~ 1 for each sample in the series. This indicates that ionic conductivity of the SBEs obeys the Arrhenius law and it is suggested that the system is thermally activated which similar to the work done by previous researchers^19^,^20^,^23^,^29^.

### FTIR Deconvolution Analysis

**Figure 2:**
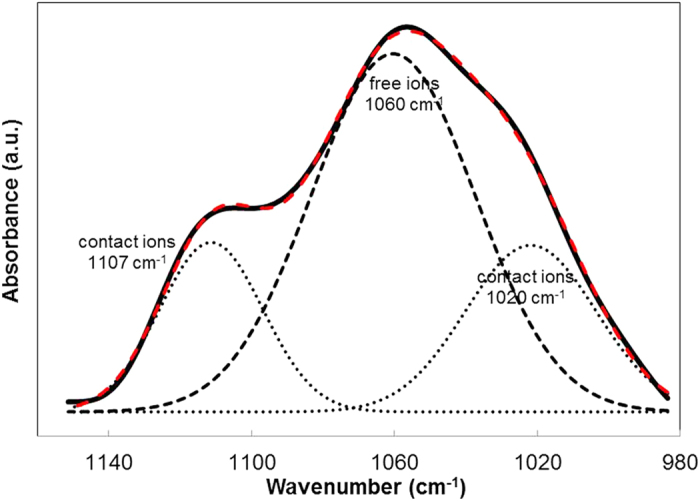
FTIR deconvolution of Gly-40.

**Figure 3:**
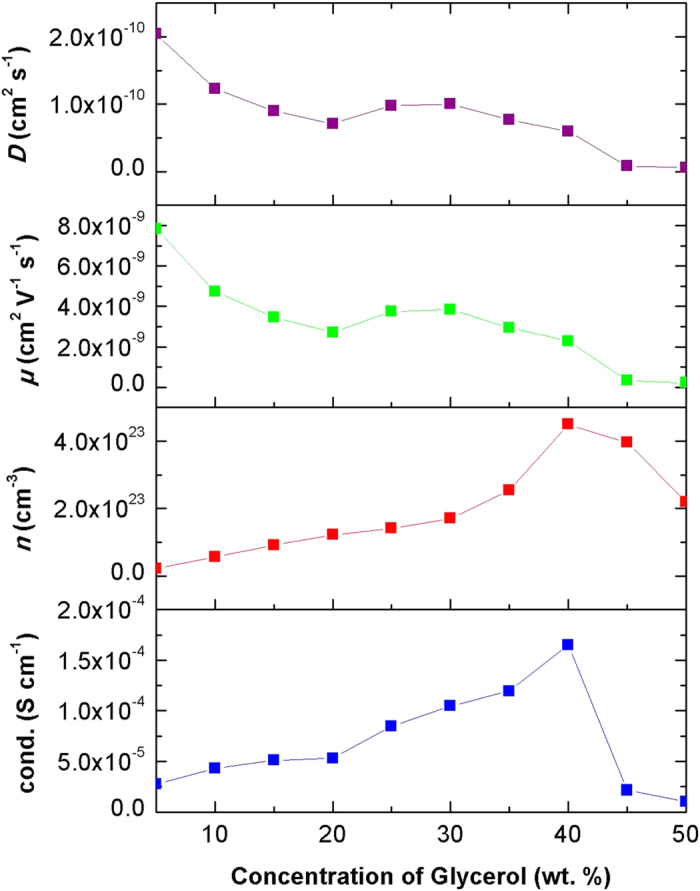
The transport parameters of the CMC-OA-Gly SBEs.

Based on the report by^33^,^34^, the band from COO^−^ anions can be observed at ~1060 cm^−1^ while contact ions appeared at 1020 cm^−1^ and 1107 cm^−1^. Hence, the wavenumbers between 1160 and 980 cm^−1^ are of interest since the bands representing the free and contact ions are within this region. FTIR deconvolution of CMC-OA-Gly SBEs was plotted and shown in Fig. 2 at the wavelength 980 cm^−1^ to 1160 cm^−1^. From Fig. 2, the peak adjacent to 1060 cm^−1^ can be assigned to free ions and the peaks adjacent to 1020 cm^−1^ and 1107 cm^−1^ can be assigned to contact ions. The percentage area of free ions and contact ions can be calculated from the ratio of the area of free or contact ions to the total area of deconvolution peaks, respectively. Table 2 lists the percentage of free ions and contact ions of the CMC-OA-Gly SBEs. Figure 3 shows the values of ***n, μ*** and ***D*** obtained.

From Table 2, it is observed that the percentage of free ions in the electrolyte increased up to the sample Gly-40. This implies that sample Gly-40 causes more ions to dissociate, thus assisting more ion conduction. Beyond sample Gly-40, the percentage of free ions was observed to decrease. This may be attributed to ion association^24^,^26^ which supported the ionic conductivity reductions of CMC-OA-Gly SBEs as showed in previous section.

From Fig. 3, it can be observed that the conductivity of CMC-OA-Gly SPEs is strongly influenced by the number density of mobile ions (*n*). The ionic mobility (*μ*) and the diffusion coefficient (*D*) plays a weak role in influencing the conductivity values of CMC-OA-Gly SBEs. As the Gly content increases, it is believed that more protons H^+^ referring to *n* are supplied due to the dissociation of plasticizer, as proved in the FTIR study from previous work^32^. The increment of *n* in the SBE systems would lead to the decrease of *E*_*a*_^25^, requiring a lower energy to move the ion due to the decrease of the values of *μ* and *D*; hence it influenced the ionic conductivity. The value of *n, μ* and *D* calculated is in reasonable agreement with that obtained by previous work^33^,^34^,^35^. Further proved of the effect by ionic mobility and diffusion coefficient was done by performing TNM.

### Ionic Transport Analysis

**Figure 4:**
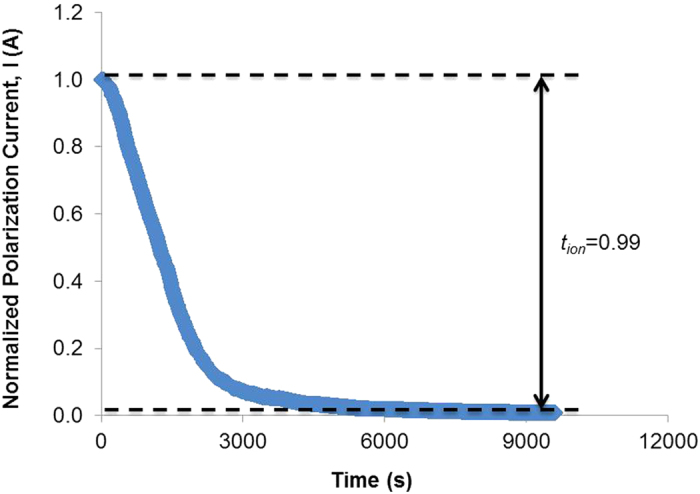
The polarized current versus time for sample Gly-40.

According to Linford^36^, in SBEs, electron conduction can be neglected. Hence measurement of the polarization current should give the cationic transference value when the polarization current saturates. Apart from this, it is necessary to know the type of conducting species ion since its mass must be known. The plot of polarized current versus time is shown in Fig. 4. From Fig. 4, it shows that the initial total current decreases with time due to the depletion of the ionic species in the electrolyte and becomes constant in the fully depleted situation. This is because, the ionic currents through an ion-blocking electrode falls rapidly with time if the electrolyte is primarily ionic. In polymer electrolytes, there are two possible mobile ionic species, i.e., cations and anions.

**Figure 5:**
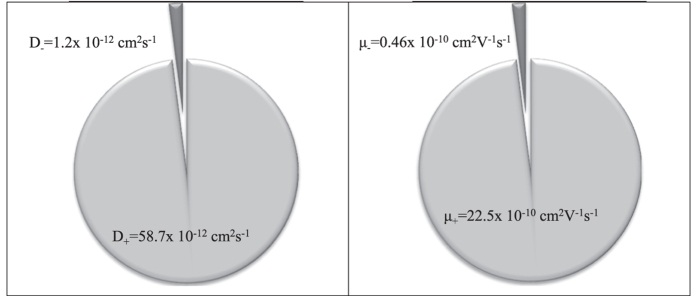
The (**a**) diffusion coefficient and (**b**) ionic mobility of cations and anions for Gly-40.

From Fig. 5, it is observed that the value of *μ*_+_ and *D*_+_ were higher than the value of *μ*_*−*_ and the *D*_*−*_. The charge transport in these CMC-OA-Gly SBE is predominantly ionic accompanied by mass transport and electronic contribution to the total current is negligible^24^,^27^,^36^. Since the CMC matrix has carboxyl groups, it can act as a good proton acceptor and provides free pathways for the proton mobility. Similar results also have been reported for different types of biopolymer electrolytes^37^,^38^,^39^.

## Conclusion

Proton conducting solid bio-polymer electrolytes based on carboxymethyl cellulose and oleic acid with different compositions of Gly as plasticizer had been prepared using solution casting techniques. The highest conductivity achieved by the CMC-OA-Gly SBEs at ambient temperature (303 K) is 1.64 × 10^−4^ S cm^−1^ for 40 wt. % of Gly. The increase in ionic conductivity is in good agreement with the increase in the number of free ions in CMC-OA-Gly SBEs. The value of *μ*_+_ and *D*_+_ were higher than the value of *μ*_*−*_ and the *D*_*−*_, thus proved that CMC-OA-Gly SBEs was a proton conductor. It can be concluded that, with the addition of Gly as plasticizer into CMC-OA system have aided the dissociation of ionic dopant, enhanced simultaneously the transport parameter and developed more proton transfer for dopant without favouring proton transfer.

## Additional Information

**How to cite this article**: Chai, M. N. and Isa, M. I. N. Novel Proton Conducting Solid Bio-polymer Electrolytes Based on Carboxymethyl Cellulose Doped with Oleic Acid and Plasticized with Glycerol. *Sci. Rep.* **6**, 27328; doi: 10.1038/srep27328 (2016).

## Figures and Tables

**Table 1:** Designation and composition of the CMC-OA-Gly SBEs.

Designation (CMC-OA-Gly)	Gly (wt%)	Gly (g)
OA-20 (control sample)^22^	0	0
Gly-5	5	0.07
Gly-10	10	0.14
Gly-15	15	0.22
Gly-20	20	0.31
Gly-25	25	0.42
Gly-30	30	0.54
Gly-35	35	0.67
Gly-40	40	0.83
Gly-45	45	1.02
Gly-50	50	1.25

**Table 2:** Percentage area of free and contact ions of the CMC-OA-Gly SBEs.

Designation	Free ions (%)	Contact ions (%)
Gly-5	37.31	62.69
Gly-10	43.48	56.52
Gly-15	44.76	55.24
Gly-20	42.00	58.00
Gly-25	36.75	63.25
Gly-30	34.79	65.21
Gly-35	41.57	58.43
Gly-40	59.90	40.10
Gly-45	43.25	56.75
Gly-50	38.15	61.85
